# Proportional Nodal Burden as a Marker of Recurrence Risk in Non-Metastatic Colorectal Adenocarcinoma: The Prognostic Role of Lymph Node Ratio

**DOI:** 10.3390/jcm15145743

**Published:** 2026-07-22

**Authors:** Adem Ozcan, Gizem Gunes

**Affiliations:** Department of Surgical Oncology, Ankara Bilkent City Hospital, 06800 Ankara, Turkey

**Keywords:** colorectal adenocarcinoma, lymph node ratio, lymph node yield, disease-free survival, overall survival, nodal staging, postoperative risk stratification, prognostic factor

## Abstract

**Background**: Pathological N staging remains central to prognostic assessment in colorectal adenocarcinoma; however, it captures nodal disease as an absolute count and does not account for the total number of examined lymph nodes. The lymph node ratio (LNR) may refine postoperative risk stratification by integrating both metastatic nodal burden and lymph node yield. This study evaluated the prognostic value of LNR in a contemporary surgical cohort of patients with colorectal adenocarcinoma. **Methods**: This retrospective single-center cohort study included consecutive adult patients who underwent surgery for histopathologically confirmed colorectal adenocarcinoma at the Surgical Oncology Clinic of Ankara Bilkent City Hospital between January 2020 and 25 March 2026. Patients with metastatic disease were excluded from the primary curative-intent analysis. LNR was calculated as the number of metastatic lymph nodes divided by the total number of harvested lymph nodes and categorized as 0, 0.01–0.20, and >0.20. Overall survival (OS) was calculated from the date of histopathological diagnosis, whereas disease-free survival (DFS) was calculated from the date of curative-intent surgery. Survival outcomes were evaluated using Kaplan–Meier analysis and Cox proportional hazards regression. **Results**: Among 309 screened patients, 274 non-metastatic patients constituted the final analytic cohort. LNR categories were LNR 0 in 159 patients (58.0%), LNR 0.01–0.20 in 76 patients (27.7%), and LNR > 0.20 in 39 patients (14.2%). Median observed follow-up was 24.1 months [IQR, 13.9–35.8] for OS and 22.4 months [IQR, 10.8–34.3] for DFS. During follow-up, 60 deaths and 90 DFS events occurred. Three-year OS was 76.4%, 71.5%, and 53.4% across the three LNR groups, respectively (log-rank *p* = 0.014), whereas 3-year DFS was 74.7%, 62.4%, and 9.6%, respectively (log-rank *p* < 0.001). In multivariable analysis, LNR > 0.20 remained independently associated with impaired DFS (adjusted HR 4.84 (95% CI 2.77–8.46); *p* < 0.001), whereas its association with OS was attenuated after adjustment. **Conclusions**: In patients undergoing surgery for non-metastatic colorectal adenocarcinoma, LNR > 0.20 identifies a high-risk subgroup with markedly impaired disease-free survival. LNR may complement conventional N staging and improve postoperative recurrence-risk stratification using information already available in routine pathology reports.

## 1. Introduction

Colorectal adenocarcinoma remains a major global oncological burden, accounting for more than 1.9 million new cases and over 900,000 deaths worldwide in 2022 [1]. Despite advances in systemic therapy, molecular stratification, and multidisciplinary treatment, curative-intent surgery with adequate oncological lymphadenectomy remains the cornerstone of treatment for non-metastatic disease. Postoperative risk stratification is still primarily based on the pathological tumor-node-metastasis system, in which nodal status has a central role because it defines stage III disease and directly influences adjuvant treatment decisions [2,3].

The prognostic value of lymph node assessment depends not only on the presence of nodal metastasis but also on the adequacy of lymph node harvest and pathological evaluation. Current surgical and oncological recommendations emphasize that at least 12 lymph nodes should be examined to confidently assign node-negative disease, and retrieval of fewer than 12 lymph nodes is considered a high-risk feature, particularly in stage II colon cancer [2,3]. However, conventional N staging is based exclusively on the absolute number of metastatic lymph nodes and does not account for the total number of harvested nodes. This limitation creates vulnerability to stage migration, especially in patients with suboptimal lymph node yield, and may reduce the discriminatory power of conventional nodal staging in routine clinical practice.

The lymph node ratio (LNR), defined as the number of metastatic lymph nodes divided by the total number of examined lymph nodes, has emerged as a practical nodal metric that integrates both tumor burden and adequacy of lymphadenectomy. Recent evidence suggests that LNR provides complementary prognostic information alongside conventional N stage, and large retrospective cohorts have identified LNR as a survival-associated nodal metric after colorectal cancer surgery [4,5]. In parallel, the log odds of positive lymph nodes (LODDS), which incorporates both positive and negative lymph node counts, has been proposed as another complementary nodal parameter; a recent systematic review and meta-analysis reported that LODDS is significantly associated with overall and disease-free survival in colorectal cancer [6]. Nevertheless, the optimal clinical use of LNR and LODDS remains unsettled because reported cut-off values vary across studies, and their comparative prognostic contribution should be interpreted within contemporary clinicopathological datasets.

Beyond anatomical stage, colorectal cancer prognosis is influenced by tumor biology, adverse pathological features, neoadjuvant response, and treatment delivery. These factors may modify recurrence risk and survival, but they do not eliminate the need for robust nodal metrics derived from routine pathology reports. Evaluating LNR within this contemporary clinicopathological context may clarify whether it adds clinically useful information without replacing conventional TNM staging.

The present study aimed to evaluate whether predefined LNR categories stratify OS and DFS in patients undergoing surgery for non-metastatic colorectal adenocarcinoma. Secondary objectives were to compare LNR with conventional N stage and LODDS and to interpret LNR within a contemporary clinicopathological framework. We hypothesized that high LNR would identify a subgroup with increased postoperative recurrence risk alongside conventional nodal classification.

## 2. Materials and Methods

### 2.1. Study Design and Setting

This study was designed as a retrospective, single-center, observational cohort study conducted at the Surgical Oncology Clinic of Ankara Bilkent City Hospital. The surgical eligibility window consisted of consecutive adult patients who underwent surgical treatment for colorectal cancer at Ankara Bilkent City Hospital between 1 January 2020, and 25 March 2026. Thus, the retrospective surgical record screening was restricted to patients treated before or on the date of ethics approval, and no patient who underwent surgery after ethics approval was included in the analytic cohort. Routine follow-up information was subsequently updated from institutional records for outcome ascertainment, and the follow-up database was locked on 31 May 2026. All analyses were performed on anonymized institutional records.

### 2.2. Ethical Approval

The study protocol was approved by the Institutional Review Board of Ankara Bilkent City Hospital (Approval No: TABED 1-26-2356, Date: 25 March 2026). The retrospective cohort screening was restricted to institutional surgical records generated before or on the date of ethics approval. No patient who underwent surgery after the ethics approval date was included in the analytic cohort. Follow-up information recorded during routine clinical care was extracted in anonymized form after approval for outcome ascertainment. The study was conducted in accordance with the principles of the Declaration of Helsinki. Because of the retrospective design, the use of anonymized hospital records, and the absence of any study-specific intervention, additional diagnostic procedure, or direct patient contact, the requirement for individual informed consent was waived by the institutional review board.

### 2.3. Patient Selection

All patients operated on for suspected or confirmed colorectal malignancy during the predefined surgical eligibility window were screened through the institutional surgical oncology operative registry. Eligibility was then confirmed by reviewing postoperative histopathology reports and hospital follow-up records.

Patients were included if they met all of the following criteria:

- age ≥18 years;
- surgical treatment for colorectal cancer at Ankara Bilkent City Hospital;
- histopathological confirmation of colorectal adenocarcinoma;
- availability of postoperative pathological lymph node assessment, including both the total number of harvested lymph nodes and the number of metastatic lymph nodes;
- availability of follow-up data sufficient to determine survival status.

Patients were excluded if they had non-adenocarcinoma histology, recurrent colorectal malignancy, incomplete pathological records, missing lymph node data, unavailable survival data, or metastatic disease at diagnosis (M1 stage). The primary analysis was restricted to patients undergoing curative-intent treatment for non-metastatic colorectal adenocarcinoma.

The final analytic cohort was established after applying these predefined inclusion and exclusion criteria. Patients operated close to the end of the surgical eligibility window were retained in descriptive and perioperative analyses and were right-censored at the date of last documented follow-up for survival analyses.

### 2.4. Data Sources and Data Extraction Workflow

Data were retrospectively obtained from the hospital information management system and institutional pathology archives using a predefined data collection framework. Clinical, operative, pathological, treatment-related, and follow-up variables were extracted from routine institutional records. Histopathological eligibility, tumor characteristics, and lymph node variables were verified from final pathology reports. LNR and LODDS were recalculated from the original lymph node counts before analysis. Internal consistency checks were performed for lymph node counts, survival status, follow-up dates, duplicated archive numbers, and biologically implausible values before the dataset was locked. All data were anonymized before statistical analysis.

### 2.5. Clinical and Demographic Variables

The following baseline clinical variables were recorded: age, sex, body mass index, American Society of Anesthesiologists score, Eastern Cooperative Oncology Group performance status, and major comorbidities.

Comorbidities included diabetes mellitus, hypertension, coronary artery disease, and chronic obstructive pulmonary disease. These variables were abstracted as binary variables according to the presence or absence of a documented diagnosis in the medical records.

### 2.6. Tumor-Related and Pathological Variables

Tumor localization was recorded according to the anatomical site documented in the operative and pathology reports. The original database categories were right colon, transverse colon, left colon, sigmoid colon, rectum, and cecum. For subgroup analyses, tumor location could also be grouped as colon versus rectum or right-sided versus left-sided colorectal cancer, depending on the statistical requirements of the analysis.

Pathological variables included tumor size, pathological T stage, pathological N stage, TNM stage, lymphovascular invasion, perineural invasion, tumor obstruction, tumor perforation, and resection margin status. Resection margin status was classified as R0 or R1 according to the final pathology report.

For patients who received neoadjuvant treatment, post-treatment pathological staging variables were recorded when available, including ypT stage, ypN stage, tumor regression grade, and pathological complete response status. These variables were analyzed only within the neoadjuvant-treatment subgroup.

### 2.7. Operative and Perioperative Variables

Operative variables included surgical approach, type of operation, operative urgency, and stoma creation.

The surgical approach was classified as open or laparoscopic. The operation type was recorded using the original operative categories: right hemicolectomy, extended right hemicolectomy, extended left hemicolectomy, left hemicolectomy, low anterior resection, anterior resection, abdominoperineal resection, total colectomy, and Hartmann procedure.

Operative urgency was classified as emergency or elective surgery. Stoma creation was recorded as absent or present according to the operative record.

### 2.8. Treatment-Related Variables

Neoadjuvant treatment status was recorded as received or not received. In patients who received neoadjuvant therapy, treatment-response variables were extracted from the postoperative pathology report where available.

Adjuvant chemotherapy exposure was recorded as a binary variable (received versus not received). Information regarding treatment completion was collected when documented in routine patient records. These treatment-related variables were primarily used to characterize the study population and were additionally explored as potential prognostic factors in survival analyses.

Because treatment variables may be influenced by stage, performance status, postoperative recovery, and physician decision-making, they were not treated as randomized exposures. Their interpretation was therefore considered observational and hypothesis-generating.

### 2.9. Molecular and Treatment-Response Variables

Mismatch repair (MMR) status was evaluated only in patients for whom immunohistochemical assessment had been performed as part of routine pathological evaluation. Expression status of MLH1, PMS2, MSH2, and MSH6 was extracted from final pathology reports. Based on the original pathology interpretation, tumors were categorized as proficient mismatch repair/microsatellite stable (pMMR/MSS) or deficient mismatch repair/microsatellite instability-high (dMMR/MSI-H). No additional immunohistochemical, molecular, or genetic testing was performed for the purposes of this study. Molecular variables were not used as primary exposure variables. Instead, they were used as exploratory clinicopathological annotations to evaluate whether the prognostic value of lymph node-based metrics differed according to tumor biology.

Tumor regression grade, ypT stage, ypN stage, and pathological complete response were evaluated only in patients who received neoadjuvant therapy and had available post-treatment pathological assessment. These analyses were restricted to the relevant subgroup to avoid inappropriate interpretation in patients who had upfront surgery.

### 2.10. Definition of Lymph Node Variables

The total lymph node yield was defined as the total number of lymph nodes harvested and examined in the surgical specimen. The positive lymph node count was defined as the number of histopathologically confirmed metastatic lymph nodes.

Patients were categorized according to total lymph node yield as:

- <12 harvested lymph nodes
- ≥12 harvested lymph nodes

The lymph node ratio was calculated for each patient as:

LNR = positive lymph nodes/total harvested lymph nodes

For categorical analyses, patients were stratified into three prespecified LNR groups:

- LNR 0
- LNR 0.01–0.20
- LNR > 0.20

These categories were selected a priori to distinguish node-negative disease, low-to-intermediate proportional nodal involvement, and high proportional nodal burden. The >0.20 threshold was chosen because it is clinically interpretable and falls within the range of high-risk LNR thresholds reported in previous colorectal cancer studies [4,5,7,8,9]. No cohort-derived optimal cut-off was generated, in order to avoid data-driven threshold selection and overestimation of prognostic performance.

The log odds of positive lymph nodes was calculated as:

LODDS = log[(positive lymph nodes + 0.5)/(negative lymph nodes + 0.5)]

where negative lymph nodes were calculated as the total number of harvested lymph nodes minus the number of positive lymph nodes. The constant 0.5 was added to allow calculation in patients with zero positive or zero negative lymph nodes.

Because pathological N stage, positive lymph node count, LNR, and LODDS are mathematically and biologically related, these variables were not forced simultaneously into the same multivariable model unless specifically justified. Separate nodal models were constructed to reduce multicollinearity and to compare the prognostic contribution of each nodal metric.

## 3. Outcomes

The primary endpoint was overall survival (OS), defined as the interval between the date of histopathological diagnosis of colorectal adenocarcinoma and death from any cause or the date of last follow-up. Patients who were alive at the end of follow-up were censored at their last documented clinical contact.

Secondary oncological endpoints included disease-free survival (DFS) and recurrence. Disease-free survival was defined as the interval between curative-intent surgical resection and the first documented recurrence, death from any cause, or last follow-up, whichever occurred first. Patients without recurrence or death were censored at the date of their last disease assessment.

Recurrence was defined as the occurrence of locoregional or distant disease relapse after curative-intent surgery, confirmed by radiological, endoscopic, operative, or pathological findings during follow-up.

Secondary perioperative endpoints included 30-day mortality, postoperative morbidity, anastomotic leakage, reoperation, and intensive care unit admission. Thirty-day mortality was defined as death from any cause within 30 days after surgery. Anastomotic leakage, reoperation, and intensive care unit admission were recorded according to documentation in the surgical and postoperative hospital records.

### 3.1. Missing Data Strategy

Missing data were assessed before statistical modeling. Variables essential for the primary analysis, including total harvested lymph node count, positive lymph node count, and survival status, were required for inclusion in the main cohort. 

Missingness was handled according to the clinical context and routine availability of each variable. MMR/MSI status was analyzed only in patients with documented immunohistochemical testing. Tumor budding was analyzed only when reported in the final pathology report. CRM distance was analyzed only in rectal cancer cases with a documented circumferential margin measurement. Tumor regression grade, ypT stage, ypN stage, and pathological complete response were analyzed only among patients who received neoadjuvant therapy and had available post-treatment pathological assessment. Adjuvant chemotherapy completion was analyzed only among patients who received adjuvant chemotherapy and had documented completion status. 

No statistical imputation was performed for the primary analysis. Variables with substantial missingness or subgroup restriction, including MMR/MSI status, tumor budding, CRM, tumor regression grade, pathological complete response, and adjuvant chemotherapy completion, were not included in the primary multivariable Cox model. These variables were evaluated as exploratory complete-case or subgroup analyses. A dedicated missing-data summary was added as Supplementary Table S1.

### 3.2. Statistical Analysis

Continuous variables were assessed for distributional characteristics using histograms, Q-Q plots, and normality tests where appropriate. Normally distributed continuous variables were presented as mean ± standard deviation, whereas non-normally distributed variables were presented as median with interquartile range. Categorical variables were presented as numbers and percentages.

Comparisons between two groups were performed using the Student’s *t*-test or Mann–Whitney U test for continuous variables, and the chi-square test or Fisher’s exact test for categorical variables, as appropriate. Comparisons among more than two groups were performed using one-way analysis of variance or the Kruskal–Wallis test according to data distribution.

Survival analyses were performed using the Kaplan–Meier method. Survival curves were compared using the log-rank test. Overall survival and disease-free survival were analyzed according to lymph node yield category, LNR category, pathological N stage, LODDS category, tumor localization, neoadjuvant treatment status, and other clinically relevant variables.

Univariable Cox proportional hazards regression analysis was used to evaluate associations between candidate variables and OS and DFS. Variables with a *p* value <0.10 in univariable analysis, together with clinically important covariates, were considered for multivariable Cox regression. Candidate variables for the final models were selected according to clinical relevance, event counts, data completeness, and avoidance of collinearity. 

The main LNR-based model included age, ECOG performance status, resection margin status, pT4 disease, lymphovascular invasion, perineural invasion, lymph node yield, and LNR category. This model contained nine regression coefficients. Given 60 OS events and 90 DFS events, the approximate events-per-variable ratio was 6.7 for OS and 10.0 for DFS. Therefore, the OS model was interpreted cautiously, and variables with substantial missingness or subgroup restriction were excluded from the primary multivariable model. 

Variables with substantial missingness or subgroup restriction, including MMR/MSI status, tumor budding, CRM, tumor regression grade, pathological complete response, and adjuvant chemotherapy completion, were not included in the primary multivariable model and were interpreted as exploratory. To minimize overfitting and multicollinearity, pathological N stage, LNR, and LODDS were evaluated in separate adjusted models rather than being entered simultaneously. 

The proportional hazards assumption was assessed using log-minus-log survival plots and Schoenfeld residual diagnostics. Model discrimination and fit were explored using Harrell’s C-index, Akaike information criterion, and likelihood ratio statistics for adjusted nodal models. A sensitivity model additionally adjusted for rectal localization and neoadjuvant treatment exposure to address potential confounding related to rectal cancer and neoadjuvant therapy. Results are reported as hazard ratios with 95% confidence intervals.

Predefined subgroup analyses were planned in node-positive patients, patients with adequate lymph node harvest, colon versus rectal cancer, patients who received neoadjuvant therapy, and patients with available MMR/MSI status. Exploratory analyses were interpreted as hypothesis-generating.

Kaplan–Meier analyses, log-rank tests, descriptive statistics, and primary Cox regression models were performed using IBM SPSS Statistics for Windows, Version 26.0 (IBM Corp., Armonk, NY, USA). Additional Cox model diagnostics and model-performance analyses, including Schoenfeld residual testing, Harrell’s C-index, Akaike information criterion, and likelihood ratio statistics, were performed using Python version 3.11 with survival-analysis packages. A two-sided *p* value <0.05 was considered statistically significant.

## 4. Results

### 4.1. Patient Flow and Study Cohort

**Figure 1:**
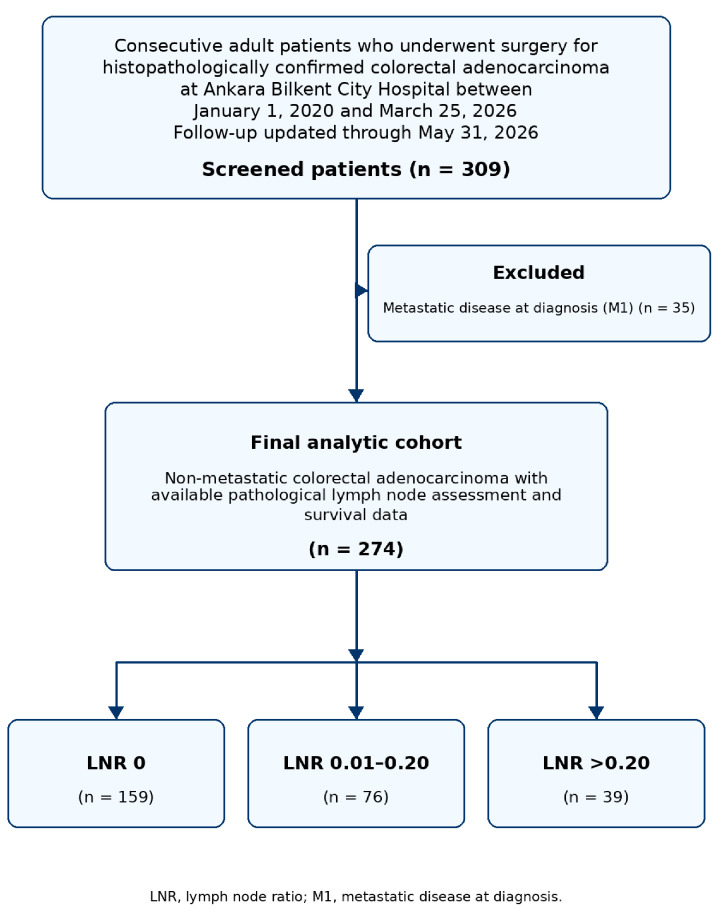
Study flow diagram. Flow diagram of the retrospective cohort. A total of 309 consecutive colorectal adenocarcinoma surgery records were screened within the predefined surgical eligibility window from January 2020 to 25 March 2026. Routine follow-up information was updated through 31 May 2026. Thirty-five patients with M1 disease were excluded from the primary curative-intent analysis. The final analytic cohort consisted of 274 patients with available pathological lymph node assessment and survival data.

A total of 309 consecutive patients who underwent surgery for histopathologically confirmed colorectal adenocarcinoma during the predefined surgical eligibility window were screened. Thirty-five patients with metastatic disease coded as M1 were excluded from the primary curative-intent analysis. The final analytic cohort therefore included 274 patients with non-metastatic colorectal adenocarcinoma who had available pathological lymph node assessment and survival data (Figure 1).

According to LNR category, 159 patients (58.0%) had an LNR of 0, 76 patients (27.7%) had an LNR of 0.01–0.20, and 39 patients (14.2%) had an LNR of >0.20.

### 4.2. Baseline and Operative Characteristics

The median age of the cohort was 64.5 years [IQR, 56.0–71.0], and 192 patients (70.1%) were male. The distribution of age, sex, ASA score, ECOG performance status, diabetes mellitus, and hypertension did not differ significantly across LNR groups (Table 1).

Rectal localization was present in 115 patients (42.0%) and did not differ significantly across LNR categories: 45.9% in the LNR 0 group, 31.6% in the LNR 0.01–0.20 group, and 46.2% in the LNR > 0.20 group (*p* = 0.097).

Most procedures were performed electively (250 patients, 91.2%), and 205 patients (74.8%) underwent open surgery. Open surgery was more frequent in the LNR > 0.20 group than in the intermediate LNR group (87.2% vs. 61.8%, overall *p* = 0.005). Stoma creation was performed in 121 patients (44.2%), without a statistically significant difference across LNR categories.

The median postoperative length of stay was 11.0 days [IQR, 8.0–14.0]. Major postoperative morbidity, defined as Clavien–Dindo grade ≥ III, occurred in 45 patients (16.4%). Anastomotic leakage, reoperation, and 30-day mortality occurred in 30 patients (10.9%), 24 patients (8.8%), and 11 patients (4.0%), respectively. These perioperative outcomes did not differ significantly across LNR groups, although 30-day mortality was numerically higher in the LNR > 0.20 group (10.3%) than in the LNR 0 and LNR 0.01–0.20 groups (3.1% and 2.6%, respectively; *p* = 0.099) (Table 1).

### 4.3. Pathological, Molecular, and Treatment-Response Characteristics

The median tumor size was 41.0 mm [IQR, 29.2–60.0]. Tumors in the LNR > 0.20 group were larger than those in the LNR 0 and LNR 0.01–0.20 groups, with median tumor sizes of 55.0 mm, 40.0 mm, and 42.5 mm, respectively (*p* = 0.010) (Table 2).

The median number of harvested lymph nodes was 17 [IQR, 11–28]. Adequate lymph node harvest, defined as ≥12 examined lymph nodes, was achieved in 201 patients (73.4%). The proportion of patients with ≥12 lymph nodes examined was highest in the LNR 0.01–0.20 group (89.5%) and lower in the LNR 0 and LNR > 0.20 groups (66.0% and 71.8%, respectively; *p* < 0.001).

Node-positive disease was present in 115 patients (42.0%). The pathological N-stage distribution was N0 in 159 patients (58.0%), N1 in 80 patients (29.2%), N2a in 16 patients (5.8%), and N2b in 19 patients (6.9%). The number of positive lymph nodes increased progressively across LNR groups, with a median positive lymph node count of 0 [IQR, 0–0] in the LNR 0 group, 2 [IQR, 1–2] in the LNR 0.01–0.20 group, and 6 [IQR, 4–9] in the LNR > 0.20 group.

Adverse pathological features were enriched in higher LNR categories. Lymphovascular invasion was present in 141 patients (51.5%) overall and increased from 29.6% in the LNR 0 group to 78.9% and 87.2% in the LNR 0.01–0.20 and LNR > 0.20 groups, respectively (*p* < 0.001). Perineural invasion was present in 105 patients (38.3%) and was also more frequent in the LNR > 0.20 group (76.9%) than in the LNR 0 and LNR 0.01–0.20 groups (28.3% and 39.5%, respectively; *p* < 0.001).

Additional pathological, treatment-response, and molecular findings are summarized in Table 2. Higher LNR categories were associated with higher tumor budding scores, shorter CRM distance in rectal cancer cases, enrichment of TRG3 among neoadjuvant-treated patients, absence of pathological complete response in node-positive LNR groups, and lower adjuvant chemotherapy completion among patients with known completion status. MMR testing was available in 161 patients, and dMMR/MSI-H status did not differ significantly across LNR categories.

### 4.4. Survival Outcomes

During follow-up, 60 deaths (21.9%) occurred in the final analytic cohort. Follow-up information was updated from routine institutional records through the database lock date of 31 May 2026. The median observed follow-up time for OS analysis was 735 days [IQR, 423–1089], corresponding to approximately 24.1 months [IQR, 13.9–35.8]. The overall Kaplan–Meier estimated OS rates were 89.5% at 1 year and 71.6% at 3 years.

**Figure 2:**
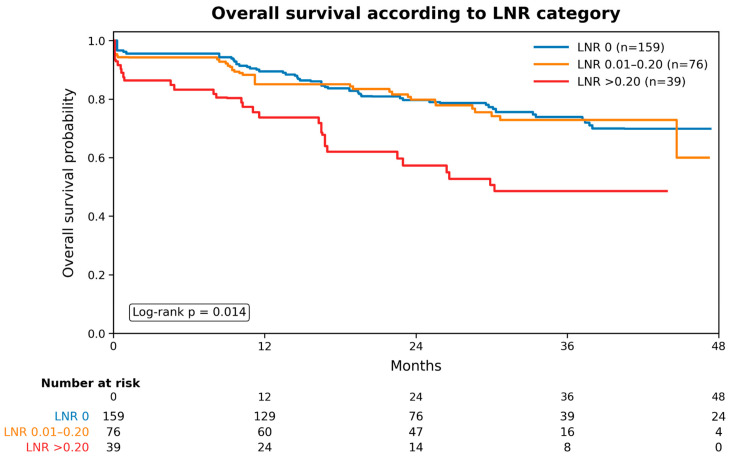
Overall survival according to LNR category. Kaplan–Meier curves for overall survival stratified by lymph node ratio category. The horizontal axis uses unified 12-month intervals from 0 to 48 months, and the number-at-risk table is aligned at 0, 12, 24, 36, and 48 months. At 36 months, the numbers at risk were 39, 16, and 8 for the LNR 0, LNR 0.01–0.20, and LNR > 0.20 groups, respectively. Overall survival differed significantly across LNR groups, with the poorest survival observed in patients with LNR > 0.20. The log-rank *p* value was calculated using the full available follow-up.

OS differed significantly across LNR groups (log-rank *p* = 0.014) (Figure 2). The 3-year OS rate was 76.4% in the LNR 0 group, 71.5% in the LNR 0.01–0.20 group, and 53.4% in the LNR > 0.20 group. At 36 months, the numbers at risk under the OS curves were 39, 16, and 8 patients in the LNR 0, LNR 0.01–0.20, and LNR > 0.20 groups, respectively. Crude mortality also increased across LNR strata, from 18.2% in the LNR 0 group to 22.4% in the LNR 0.01–0.20 group and 35.9% in the LNR > 0.20 group.

A total of 90 DFS events (32.8%) occurred. Documented recurrence was observed in 51 patients (18.6%), and 39 additional DFS events were deaths without documented recurrence. The median observed follow-up time for DFS analysis was 683 days [IQR, 329–1044], corresponding to approximately 22.4 months [IQR, 10.8–34.3]. The overall Kaplan–Meier estimated DFS rates were 79.7% at 1 year and 60.4% at 3 years.

**Figure 3:**
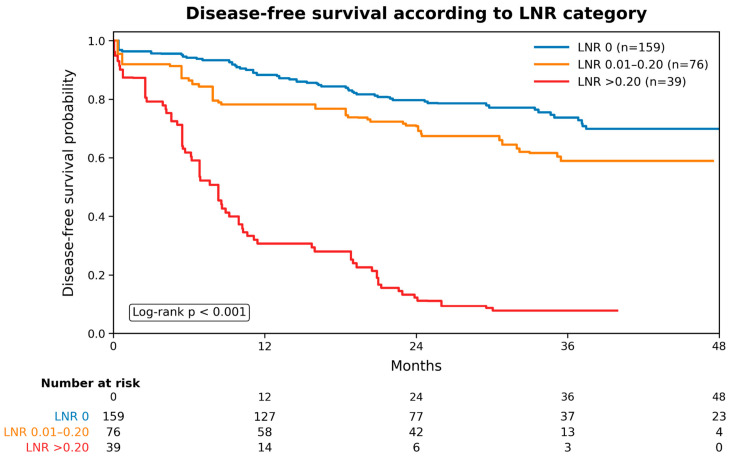
Disease-free survival according to LNR category. Kaplan–Meier curves for disease-free survival stratified by lymph node ratio category. The horizontal axis uses unified 12-month intervals from 0 to 48 months, and the number-at-risk table is aligned at 0, 12, 24, 36, and 48 months. At 36 months, the numbers at risk were 37, 13, and 3 for the LNR 0, LNR 0.01–0.20, and LNR > 0.20 groups, respectively. DFS decreased stepwise with increasing LNR, and patients with LNR > 0.20 had markedly inferior DFS compared with the other groups. The log-rank *p* value was calculated using the full available follow-up.

DFS showed a strong stepwise deterioration across LNR categories (log-rank *p* < 0.001) (Figure 3). The 3-year DFS rate was 74.7% in the LNR 0 group, 62.4% in the LNR 0.01–0.20 group, and 9.6% in the LNR > 0.20 group. At 36 months, the numbers at risk under the DFS curves were 37, 13, and 3 patients in the LNR 0, LNR 0.01–0.20, and LNR > 0.20 groups, respectively. The LNR > 0.20 group had a particularly high DFS event burden, with 35 of 39 patients (89.7%) experiencing recurrence or death. These 35 DFS events consisted of 31 documented recurrences and 4 deaths without documented recurrence. Among the 31 patients with documented recurrence in the LNR > 0.20 group, 10 subsequently died. Because recurrence-site data were not uniformly available in structured records, site-specific recurrence patterns were not analyzed.

When conventional N stage was evaluated, N2 disease was associated with markedly worse survival. The 3-year OS rate was 76.4% in N0, 73.2% in N1, and 44.5% in N2 disease (log-rank *p* < 0.001). The corresponding 3-year DFS rates were 74.7%, 57.4%, and 9.4%, respectively (log-rank *p* < 0.001). In contrast, lymph node yield <12 versus ≥12 was not significantly associated with OS or DFS in Kaplan–Meier analysis (*p* = 0.637 and *p* = 0.494, respectively) (Table 3).

### 4.5. Cox Regression Analyses

In univariable Cox regression analysis, LNR > 0.20 was associated with worse OS compared with LNR 0 (HR 2.49 (95% CI 1.31–4.74); *p* = 0.005) and with markedly worse DFS (HR 8.27 (95% CI 5.05–13.54); *p* < 0.001).

In the LNR-based multivariable Cox model adjusted for age, ECOG performance status, resection margin status, pT4 disease, lymphovascular invasion, perineural invasion, and lymph node yield, LNR > 0.20 remained independently associated with DFS (adjusted HR 4.84 (95% CI 2.77–8.46); *p* < 0.001). Its association with OS was attenuated after adjustment and did not reach statistical significance (adjusted HR 1.77 (95% CI 0.84–3.71); *p* = 0.132) (Table 4). Schoenfeld residual diagnostics showed no evidence of proportional hazards violation for the main exposure LNR > 0.20 in either the OS model (*p* = 0.273) or the DFS model (*p* = 0.460).

In the same adjusted model, independent predictors of worse OS were ECOG ≥2 (HR 2.52 (95% CI 1.38–4.62); *p* = 0.003), R1 resection (HR 2.41 (95% CI 1.17–4.94); *p* = 0.016), and pT4 disease (HR 1.85 (95% CI 1.00–3.42); *p* = 0.049). For DFS, independent adverse predictors included ECOG ≥2 (HR 3.07 (95% CI 1.83–5.16); *p* < 0.001), R1 resection (HR 2.18 (95% CI 1.22–3.91); *p* = 0.009), pT4 disease (HR 1.87 (95% CI 1.15–3.06); *p* = 0.012), lymphovascular invasion (HR 2.02 (95% CI 1.10–3.68); *p* = 0.022), and LNR > 0.20.

Because N stage, LNR, and LODDS are mathematically related nodal variables, they were evaluated in separate adjusted models. In the conventional N-stage model, N2 disease remained independently associated with both OS (HR 2.62 (95% CI 1.24–5.55); *p* = 0.012) and DFS (HR 7.01 (95% CI 3.85–12.78); *p* < 0.001). In the LODDS-based model, LODDS was independently associated with DFS (HR 1.47 per 1-unit increase (95% CI 1.21–1.79); *p* < 0.001) but not with OS (HR 0.99 (95% CI 0.77–1.26); *p* = 0.914) (Table 4).

To address potential confounding by rectal cancer and neoadjuvant treatment, an exploratory sensitivity model additionally adjusted for rectal localization and neoadjuvant treatment exposure. In this model, LNR > 0.20 remained independently associated with impaired DFS (adjusted HR 5.31 (95% CI 2.95–9.55); *p* < 0.001), whereas its association with OS remained non-significant (adjusted HR 1.93 (95% CI 0.90–4.13); *p* = 0.091). In the LNR > 0.20 group, DFS events occurred in 20 of 21 colon cancer patients and 15 of 18 rectal cancer patients; by neoadjuvant treatment exposure, DFS events occurred in 23 of 24 non-neoadjuvant-treated patients and 12 of 15 neoadjuvant-treated patients. 

Adjuvant chemotherapy completion was explored descriptively because it was a post-baseline treatment-delivery variable with incomplete documentation and potential reverse-causation bias. Among 210 patients who received adjuvant chemotherapy, completion status was documented in 153 patients. In the LNR > 0.20 group, DFS events occurred in 18 of 21 patients with known completion status, including 6 of 9 patients who completed adjuvant chemotherapy and 12 of 12 patients who did not complete adjuvant chemotherapy. Because of small subgroup numbers, missing completion data, and the post-baseline nature of treatment completion, formal causal or interaction modeling was not used for the primary inference.

Model performance comparisons are summarized in Supplementary Table S2. For DFS, the adjusted C-index values were 0.814 for the LNR model, 0.821 for the N-stage model, and 0.791 for the LODDS model. The corresponding AIC values were 835.75, 827.05, and 851.20, respectively. For OS, the adjusted C-index values were 0.733 for the LNR model, 0.753 for the N-stage model, and 0.718 for the LODDS model.

## 5. Discussion

In this single-center retrospective cohort of patients undergoing surgery for non-metastatic colorectal adenocarcinoma, LNR > 0.20 identified a clinically distinct high-risk subgroup with markedly impaired DFS. The association with DFS remained independent after multivariable adjustment, whereas the association with OS was attenuated after adjustment for non-nodal clinical and pathological factors. These findings support interpretation of LNR as a clinically interpretable marker of proportional nodal burden and postoperative recurrence risk, used as an adjunct to conventional TNM staging rather than as a replacement.

Conventional N staging remains indispensable, but it captures only the absolute number of metastatic nodes and does not account for the total number of examined nodes. LNR addresses this limitation by incorporating both the extent of nodal metastasis and the adequacy of nodal assessment. The present findings are consistent with prior studies and meta-analyses showing that ratio-based nodal metrics provide additional prognostic information in colorectal cancer [4,5,7,10,11].

The prognostic effect of LNR was more pronounced for DFS than for OS. DFS is more directly linked to tumor biology, residual microscopic disease, surgical radicality, and early recurrence after curative-intent resection, whereas OS is influenced by age, comorbidity, performance status, subsequent therapy, treatment completion, salvage surgery, and non-cancer mortality. In this cohort, the unadjusted OS association of LNR > 0.20 was partly explained by non-nodal factors, while the DFS association persisted after adjustment. Therefore, the most defensible interpretation is that LNR is particularly useful for identifying patients at high risk of recurrence.

The relatively limited and heterogeneous follow-up duration should also be considered when interpreting the OS findings. Although the surgical eligibility window ended on the ethics approval date, follow-up information was updated from routine institutional records through the database lock date of 31 May 2026. Patients treated close to the end of the surgical eligibility window contributed shorter observation time and were right-censored at the last documented follow-up. This may partly explain why the crude OS separation across LNR groups was attenuated after multivariable adjustment, whereas the DFS association remained robust. Accordingly, the DFS findings should be regarded as the more mature oncological signal in this cohort, whereas longer follow-up is required to determine whether the observed recurrence-risk gradient translates into a sustained OS difference.

The absence of a significant survival difference according to lymph node yield <12 versus ≥12 should not be interpreted as undermining the importance of adequate nodal harvest. Examination of at least 12 lymph nodes remains a key quality and staging benchmark in colorectal cancer management [2,3]. Several factors may explain why this threshold did not stratify OS or DFS in the present cohort. First, the ≥12-node threshold primarily reflects staging adequacy rather than proportional metastatic tumor burden. Second, 73.4% of patients had at least 12 nodes examined, which may have reduced the discriminatory contrast between yield categories. Third, lymph node yield is influenced by tumor location, immune response, surgical technique, pathological processing, patient-related factors, and neoadjuvant treatment exposure. Finally, once proportional nodal involvement was captured by LNR, lymph node yield alone did not retain independent prognostic value. Therefore, our findings support the continued use of ≥12 nodes as a quality benchmark, while suggesting that ratio-based nodal metrics may provide more clinically relevant recurrence-risk information after adequate staging has been achieved [11,12].

The comparative analysis of LNR, conventional N stage, and LODDS provided a more nuanced interpretation of nodal prognostication. N stage remains indispensable and, in the present dataset, the N-stage model showed numerically favorable model performance compared with the LNR and LODDS models. This finding argues against interpreting LNR as a replacement for TNM staging. Rather, LNR should be viewed as a complementary metric that expresses proportional metastatic nodal burden in a clinically intuitive way. LODDS also incorporates both positive and negative lymph node counts and may be useful in extreme nodal-count situations, especially when the positive-node count is zero or when few negative nodes are retrieved. In our cohort, both LNR and LODDS were more strongly associated with DFS than with OS, supporting the interpretation that proportional nodal metrics are particularly relevant to recurrence-related outcomes. The practical advantage of LNR is that it defines a discrete and easily interpretable high-risk subgroup: patients with more than one fifth of examined lymph nodes involved had markedly impaired DFS.

Higher LNR was accompanied by adverse pathological features, including lymphovascular invasion, perineural invasion, higher tumor budding scores, and shorter CRM distance in rectal cancer cases. These associations support the biological plausibility of the LNR signal but should be interpreted as exploratory because tumor budding and CRM data were not available for all patients.

The neoadjuvant-treated subgroup also requires cautious interpretation. TRG3 was enriched and pathological complete response was absent in node-positive LNR groups, suggesting that residual nodal disease after neoadjuvant treatment may reflect treatment-resistant biology. Because neoadjuvant treatment can reduce lymph node yield, alter nodal positivity, and shift staging toward yp-based categories, we performed an exploratory sensitivity model additionally adjusted for rectal localization and neoadjuvant treatment. LNR > 0.20 remained associated with impaired DFS in this model, but subgroup-specific colon and rectal cut-offs require validation in larger cohorts.

MMR/MSI status was available only in a subset and was used as an exploratory clinicopathological annotation. No definitive inference regarding interaction between MMR/MSI status and LNR should be drawn from these data.

Adjuvant chemotherapy completion was lower in the LNR > 0.20 group among patients with known completion status. This variable is clinically important but methodologically complex because treatment completion occurs after surgery and may be influenced by postoperative recovery, performance status, early recurrence, treatment toxicity, physician decision-making, and patient preference. Therefore, chemotherapy completion was not included in the primary multivariable model as a causal baseline covariate. Exploratory event counts suggested that non-completion may have contributed to the poor DFS observed in the LNR > 0.20 group, but the high DFS event burden was also present among high-LNR patients who completed adjuvant chemotherapy. Accordingly, the observed DFS association should not be interpreted as a purely biological effect of LNR independent of postoperative treatment delivery. These findings support interpreting high LNR as a marker of a clinically vulnerable postoperative subgroup, while avoiding causal claims regarding treatment completion.

Clinically, LNR is attractive because it is reproducible, inexpensive, and available from routine pathology reports. Its role should be as an adjunct to, not a substitute for, TNM staging. In postoperative multidisciplinary assessment, LNR may be interpreted alongside T stage, N stage, resection margin, LVI, PNI, neoadjuvant response, MMR/MSI status, and treatment fitness.

This study has several strengths. First, it reflects a consecutive real-world surgical oncology cohort from a high-volume tertiary center. Second, the primary analysis was restricted to non-metastatic, curative-intent patients, preserving the clinical interpretability of OS and DFS. Third, nodal metrics were recalculated from raw pathological counts. Fourth, LNR was contextualized against conventional N stage, LODDS, pathological risk factors, neoadjuvant response variables, MMR/MSI status, and treatment-related variables.

Several limitations must also be acknowledged. The retrospective single-center design introduces risks of selection bias, information bias, and unmeasured confounding, and institutional patterns of surgery, pathology processing, neoadjuvant treatment, adjuvant treatment delivery, and follow-up may limit generalizability. Although the cohort was consecutive, residual confounding by rectal cancer biology, neoadjuvant treatment, treatment response, and postoperative systemic therapy cannot be fully excluded.

Follow-up duration was limited and heterogeneous because the surgical eligibility window extended from January 2020 to 25 March 2026, and follow-up information was updated through 31 May 2026. Patients treated close to the end of the surgical eligibility window contributed shorter observation time. This limitation is particularly relevant for OS analyses because longer-term cancer-related and non-cancer mortality may not yet be fully captured, and the attenuation of the OS association should not be interpreted as definitive evidence that LNR lacks long-term survival relevance. The 3-year DFS estimate in the LNR > 0.20 group was based on a small number at risk at 36 months and should therefore be interpreted as a descriptive high-risk signal requiring validation.

MMR/MSI, tumor budding, CRM, adjuvant chemotherapy completion, and neoadjuvant response variables were incompletely available or subgroup-restricted and were therefore analyzed only as exploratory complete-case or subgroup variables. In particular, because adjuvant chemotherapy completion was incompletely documented and occurred after surgery, the present data cannot fully disentangle the prognostic contribution of LNR from potential treatment-delivery confounding. Recurrence-site data were not uniformly available in structured records, preventing a robust analysis of locoregional versus distant recurrence patterns. The number of OS events limited the complexity of multivariable modeling, and overfitting was mitigated by limiting covariate selection, reporting events-per-variable considerations, and constructing separate nodal models rather than forcing N stage, LNR, and LODDS into the same model. Finally, the LNR cut-off used in this study is clinically interpretable and literature-supported, but it requires external validation before being adopted as a formal decision threshold.

## 6. Conclusions

In this retrospective cohort of patients undergoing surgery for non-metastatic colorectal adenocarcinoma, LNR > 0.20 identified a clinically relevant high-risk subgroup with markedly impaired disease-free survival. The prognostic effect of LNR was strongest for recurrence-related outcomes and remained significant in the primary adjusted model, whereas its association with overall survival was attenuated after adjustment. Given the incomplete documentation and post-baseline nature of adjuvant chemotherapy completion, this finding should not be interpreted as evidence of a purely biological effect of LNR independent of treatment delivery. LNR should not replace conventional TNM staging, but it may complement postoperative recurrence-risk stratification by incorporating proportional nodal metastatic burden into routine multidisciplinary assessment. External validation in larger multicenter cohorts with longer follow-up is required before the >0.20 threshold can be adopted as a formal decision-making cut-off.

## Figures and Tables

**Table 1:** Baseline and perioperative characteristics according to LNR category.

Variable	Overall n = 274	LNR 0 n = 159	LNR 0.01–0.20 n = 76	LNR > 0.20 n = 39	*p* Value
Age, years	64.5 [56.0–71.0]	66.0 [55.0–71.0]	64.0 [55.5–69.5]	64.0 [59.0–74.5]	0.936
Male sex	192 (70.1)	107 (67.3)	53 (69.7)	32 (82.1)	0.196
ASA ≥ 3	48 (17.5)	28 (17.6)	12 (15.8)	8 (20.5)	0.819
ECOG ≥ 2	88 (32.1)	50 (31.4)	22 (28.9)	16 (41.0)	0.406
Diabetes mellitus	85 (31.0)	52 (32.7)	24 (31.6)	9 (23.1)	0.504
Hypertension	107 (39.1)	59 (37.1)	35 (46.1)	13 (33.3)	0.308
Rectal localization	115 (42.0)	73 (45.9)	24 (31.6)	18 (46.2)	0.097
Emergency surgery	24 (8.8)	14 (8.8)	6 (7.9)	4 (10.3)	0.914
Open surgery	205 (74.8)	124 (78.0)	47 (61.8)	34 (87.2)	0.005
Stoma creation	121 (44.2)	76 (47.8)	26 (34.2)	19 (48.7)	0.120
Length of stay, days	11.0 [8.0–14.0]	10.0 [7.0–14.0]	11.0 [8.0–15.0]	11.0 [7.0–14.5]	0.564
Clavien–Dindo ≥ III	45 (16.4)	23 (14.5)	18 (23.7)	4 (10.3)	0.108
Anastomotic leakage	30 (10.9)	14 (8.8)	10 (13.2)	6 (15.4)	0.383
Reoperation	24 (8.8)	13 (8.2)	7 (9.2)	4 (10.3)	0.906
30-day mortality	11 (4.0)	5 (3.1)	2 (2.6)	4 (10.3)	0.099

**Table 2:** Pathological, molecular, and treatment-response characteristics according to LNR category.

Variable	Overall n = 274	LNR 0 n = 159	LNR 0.01–0.20 n = 76	LNR > 0.20 n = 39	*p* Value
Tumor size, mm	41.0 [29.2–60.0]	40.0 [25.0–56.0]	42.5 [30.8–56.2]	55.0 [35.0–71.5]	0.010
pT4 disease	56 (20.4)	31 (19.5)	15 (19.7)	10 (25.6)	0.684
Harvested lymph nodes	17 [11–28]	16 [9–26]	20 [15–29]	18 [11–31]	0.010
LN yield ≥ 12	201 (73.4)	105 (66.0)	68 (89.5)	28 (71.8)	<0.001
Positive lymph nodes	0 [0–2]	0 [0–0]	2 [1–2]	6 [4–9]	descriptive
LODDS	−2.83 [−3.56–−1.85]	−3.50 [−3.97–−2.94]	−2.05 [−2.40–−1.75]	−0.51 [−0.89–−0.25]	descriptive
R1 resection	23 (8.4)	10 (6.3)	6 (7.9)	7 (17.9)	0.062
Lymphovascular invasion	141 (51.5)	47 (29.6)	60 (78.9)	34 (87.2)	<0.001
Perineural invasion	105 (38.3)	45 (28.3)	30 (39.5)	30 (76.9)	<0.001
Tumor budding score	4.0 [2.0–7.0]; n = 215	2.0 [1.0–4.0]; n = 131	6.0 [4.2–7.0]; n = 58	8.5 [6.0–9.0]; n = 26	<0.001
CRM, rectal cases, mm	8.5 [1.6–9.4]; n = 99	8.9 [8.1–9.7]; n = 69	1.7 [1.5–8.3]; n = 15	1.3 [1.2–1.7]; n = 15	<0.001
Neoadjuvant treatment	103 (37.6)	67 (42.1)	21 (27.6)	15 (38.5)	0.099
TRG3 among neoadjuvant-treated	18/103 (17.5)	5/67 (7.5)	3/21 (14.3)	10/15 (66.7)	<0.001
pCR among neoadjuvant-treated	25/103 (24.3)	25/67 (37.3)	0/21 (0.0)	0/15 (0.0)	<0.001
Adjuvant chemotherapy received	210 (76.6)	111 (69.8)	66 (86.8)	33 (84.6)	0.007
Adjuvant chemotherapy completed among known	129/153 (84.3)	66/77 (85.7)	54/55 (98.2)	9/21 (42.9)	<0.001
MMR tested	161 (58.8)	101 (63.5)	45 (59.2)	15 (38.5)	0.017
dMMR/MSI-H among tested	31 (19.3)	24 (23.8)	4 (8.9)	3 (20.0)	0.109

**Table 3:** Survival outcomes according to nodal categories.

Category	n	OS Events	1-Year OS	3-Year OS	DFS Events	1-Year DFS	3-Year DFS
Overall	274	60	89.5%	71.6%	90	79.7%	60.4%
LNR 0	159	29	92.6%	76.4%	31	90.6%	74.7%
LNR 0.01–0.20	76	17	88.8%	71.5%	24	81.1%	62.4%
LNR > 0.20	39	14	78.4%	53.4%	35	35.9%	9.6%
N0	159	29	92.6%	76.4%	31	90.6%	74.7%
N1	80	17	90.9%	73.2%	29	82.3%	57.4%
N2	35	14	71.4%	44.5%	30	24.9%	9.4%
LN yield < 12	73	15	91.1%	72.5%	22	85.4%	60.0%
LN yield ≥ 12	201	45	89.0%	71.3%	68	77.7%	60.8%

**Table 4:** Multivariable Cox regression analyses.

(**A**) Main LNR-Based Adjusted Model.		
**Variable**	**Adjusted HR (95% CI) for OS**	**Adjusted HR (95% CI) for DFS**
Age, per 10 years	1.14 (0.87–1.49); *p* = 0.331	0.88 (0.71–1.08); *p* = 0.215
ECOG ≥ 2	2.52 (1.38–4.62); *p* = 0.003	3.07 (1.83–5.16); *p* < 0.001
R1 resection	2.41 (1.17–4.94); *p* = 0.016	2.18 (1.22–3.91); *p* = 0.009
pT4 disease	1.85 (1.00–3.42); *p* = 0.049	1.87 (1.15–3.06); *p* = 0.012
Lymphovascular invasion	1.28 (0.63–2.62); *p* = 0.496	2.02 (1.10–3.68); *p* = 0.022
Perineural invasion	1.21 (0.65–2.25); *p* = 0.557	1.56 (0.93–2.62); *p* = 0.090
LN yield ≥ 12	0.85 (0.45–1.60); *p* = 0.611	0.83 (0.49–1.42); *p* = 0.504
LNR 0.01–0.20 vs. 0	1.04 (0.51–2.13); *p* = 0.905	1.21 (0.65–2.24); *p* = 0.552
LNR > 0.20 vs. 0	1.77 (0.84–3.71); *p* = 0.132	4.84 (2.77–8.46); *p* < 0.001
(**B**) Alternative nodal metrics entered in separate adjusted models.		
**Nodal Metric**	**Adjusted HR (95% CI) for OS**	**Adjusted HR (95% CI) for DFS**
N1 vs. N0	0.89 (0.44–1.80); *p* = 0.751	1.33 (0.75–2.37); *p* = 0.335
N2 vs. N0	2.62 (1.24–5.55); *p* = 0.012	7.01 (3.85–12.78); *p* < 0.001
LODDS, per 1-unit increase	0.99 (0.77–1.26); *p* = 0.914	1.47 (1.21–1.79); *p* < 0.001

## Data Availability

The datasets generated and/or analyzed during the current study are available from the corresponding author on reasonable request.

## Supplementary Materials

The following supporting information can be downloaded at: https://www.mdpi.com/article/10.3390/jcm15145743/s1, Table S1: Missing data summary for variables with incomplete or subgroup-restricted availability; Table S2: Model performance comparison for adjusted nodal models.
